# Vitamin B6 Alleviates Lipopolysaccharide-induced Myocardial Injury by Ferroptosis and Apoptosis Regulation

**DOI:** 10.3389/fphar.2021.766820

**Published:** 2021-12-24

**Authors:** Meirong Shan, Xujie Yu, Yajie Li, Changning Fu, Cheng Zhang

**Affiliations:** ^1^ The Key Laboratory of Cardiovascular Remodeling and Function Research, The State and Shandong Province Joint Key Laboratory of Translational Cardiovascular Medicine, Chinese Ministry of Education, Chinese National Health Commission and Chinese Academy of Medical Sciences, Qilu Hospital of Shandong University, Jinan, China; ^2^ Department of Geriatrics, The First Affiliated Hospital of Zhengzhou University, Zhengzhou, China; ^3^ Department of Gynecology and Obstetrics, Union Hospital, Tongji Medical College, Huazhong University of Science and Technology, Wuhan, China; ^4^ Department of Critical Care Medicine, Shandong Provincial Hospital Affiliated to Shandong First Medical University, Jinan, China

**Keywords:** vitamin B6, lipopolysaccharide, myocardial injury, ferroptosis, apoptosis, Nrf2

## Abstract

Vitamin B6 (VitB6) is a water-soluble vitamin and includes pyridoxine, pyridoxal, pyridoxamine, and their phosphorylated forms. In the current study, we demonstrated that VitB6 could improve lipopolysaccharide (LPS)–induced myocardial injury. We demonstrated that VitB6 can suppress LPS-induced oxidative stress and lipid peroxidation that lead to ferroptosis and apoptosis *in vivo* and *in vitro*. Moreover, we found that VitB6 can regulate the expression of iron regulatory proteins, maintaining intracellular iron homeostasis. To confirm that VitB6 could inhibit LPS-induced ferroptosis and apoptosis, we pretreated mice with ferrostatin-1 (Fer-1) and emricasan that efficiently mimicked VitB6 pharmacological effects. This improved the survival rate of mice challenged with a high LPS dose. In addition, VitB6 regulated the expression of LPS-induced apoptosis-related proteins and iron regulatory proteins. It mediated the expression of Nrf2, transcription factor NF-E2–related factor 2, which promoted the expression of antioxidant enzymes and restrained LPS-induced ferroptosis and apoptosis. Overall, our results indicated that VitB6 can be used on novel therapies to relieve LPS-induced myocardial injury.

## Introduction

Vitamin B6 (VitB6), such as pyridoxine, pyridoxal, pyridoxamine, and pyridoxal 5′-phosphate (PLP) (the biologically active form of VitB6), is a pivotal cofactor of more than 100 enzymes (Benabdellah et al., 2009). VitB6 is important in the deamination and transamination of amino acids, gluconeogenic metabolism, ornithine cycle, and heme biosynthesis (Benabdellah et al., 2009). Heme synthesis will be blocked when VitB6 is deficient or depleted, resulting in low-pigment small-cell anemia and iron elevation (Yasuda et al., 2015). Recently, it has been indicated that VitB6 can prevent lipid peroxidation and oxygen radical production, induced by hydrogen peroxide (Kannan and Jain, 2004; Molina-López et al., 2016). Besides, VitB6 could prevent oxidative stress caused by homocysteine (Hsu et al., 2015).

Systemic sepsis causes multiple-organ dysfunction or failure (Singer et al., 2016), and the main death causes for patients in intensive care units are severe sepsis and septic shock (Calis et al., 2013). Although broad-spectrum antibiotics are widely applied in the clinic, sepsis remains an insurmountable problem (Gaieski et al., 2013). The overproduction of sepsis-induced inflammatory cytokines can also trigger heart failure in patients (Marchant et al., 2012). The inflammatory injury process includes structural and functional deficits (Calis et al., 2013). Lipopolysaccharide (LPS), a main bioactive component of the Gram-negative bacteria cell wall, is crucial to initiate inflammatory cascade responses (Simpson and Trent, 2019). Many studies have demonstrated that the structure and function of LPS-induced myocardial injury are distinctly depressed (Suzuki et al., 2003; Tzanavari et al., 2016).

Research studies have shown that LPS can result in different cell death types, including apoptosis, necrosis, and autophagy. Recently, studies have indicated that multiple mechanisms participate in cell death regulation. Currently, apoptosis, necrosis, and autophagy are well-known phenomena. Ferroptosis comprehends a new cell death type, distinct from apoptosis, necrosis, and autophagy in genetical, biochemical, and morphological aspects (Dixon et al., 2012). This iron-dependent cell death type is characterized by the accumulation of intracellular reactive oxygen species (ROS) and lipid peroxidation products (Dixon et al., 2012; Li et al., 2021). Many researchers demonstrated that ferroptosis can be related to multiple diseases, such as ischemia-reperfusion (I/R)–induced cardiomyopathy (Fang et al., 2019), kidney degeneration, neurodegenerative diseases (Stockwell et al., 2017), and various cancers. Hence, inhibiting or enhancing ferroptosis might be an emerging treatment strategy for relevant human diseases. Apoptosis, a programmed cell death form, can be initiated by physiological and pathological stimulation (Kerr et al., 1972). In the intrinsic apoptotic pathway, various stimuli can lead to the disequilibrium of pro- and anti-apoptotic Bcl2 family proteins (Paone et al., 2019). On the other hand, the extrinsic apoptotic pathway is related to caspase activation (Paone et al., 2019). Finally, emerging studies indicated that abnormal apoptosis is associated with different diseases.

In the current study, we investigated the effects of VitB6 on LPS-induced myocardial injury and its role on ferroptosis and apoptosis *in vivo* and *in vitro*.

## Materials and Methods

The full description of the materials and methods used in this study, including reagents, cell cultures, cardiac troponin I (cTnI), lactic dehydrogenase (LDH), ROS, malondialdehyde (MDA), and superoxide dismutase (SOD) measurements, animal experimental protocol, *in vitro* and *in vivo* LPS challenges, HE staining, and Western blot, can be found in the Supplementary Material.

### Animals and Protocols

Male c57BL/6 mice (8 weeks old) were purchased from Beijing Wei Tong Li Hua Experimental Animal Technology Co. Ltd. (Beijing, China). Mice were fed in temperature-controlled cages with a 12-h light-dark cycle and received freely normal chow and water, as previously described (Ma et al., 2017). This study was carried out in strict accordance with the recommendations in the Guide for the Care and Use of Laboratory Animals of the National Institutes of Health. The animal protocol was reviewed and approved by the University of Shandong Animal Care and Use Committee.

### *In vivo* LPS Challenge

Three age-matched male WT mice cohorts received an intraperitoneal injection with *Salmonella typhosa* LPS in PBS, with or without VitB6 pre-treatment (20 mg/kg, 6 h) at 4 mg/kg for 24 h (Supplementary Figure S3A).

### Cardiac Function Measurement

Mice were divided into control (*n* = 8), LPS (*n* = 9), and VitB6+LPS (*n* = 9) groups. Mice were pretreated with PBS or VitB6 for 6 h and then treated with LPS (4 mg/kg) for 24 h. Cardiac ultrasound was performed before sacrifice. Inhaled isoflurane was given to mice for volatile anesthesia and the chest hair was removed with a depilatory cream. Then, mice were fixed on the warmed imaging platform and wore with the coupling agent. The Vevo2100 imaging system, equipped with a 40-MHz high-frequency transducer (VisualSonics Inc., Toronto, Canada), was applied to perform non-invasive examinations. The M-mode echocardiogram at the parasternal long axis was used to obtain the ejection fraction (EF) of left ventricular and fractional shortening (FS).

### Measurement of cTnI, LDH, ROS, MDA, and SOD

The levels of cTnI, LDH, MDA, and SOD in serum, as well as MDA and SOD concentrations in the myocardial tissue, were detected using different kits. Total and lipid ROS were assayed by flow cytometry.

### Western Blot Analyses

Protein levels in H9C2 cell lysates and myocardial tissue homogenates were analyzed by Western blot. BCA protein assay kit was applied to determine protein concentrations. Briefly, 20 µg of protein samples were separated by 10% SDS-PAGE and then electroblotted onto polyvinylidene fluoride membranes. The membrane was blocked in 5% bull serum albumin for 1.5–2 h at room temperature, then washed three times by TBST, and submerged in diluted primary antibodies (1:1,000) overnight at 4°C, followed by the secondary antibodies for 2 h at room temperature. Finally, protein-attached bands were visualized using the ECL chemiluminescence system (Millipore Corp. MA, United States).

### Survival Measurement

A cosolvent, composed of DMSO, Tween 80, NMP, and PEG400 in different proportions, with or without ferrostatin-1 (Fer-1; 2 mg/kg), emricasan (2.5 mg/kg), and VitB6 (20 mg/kg) were given to mice by i.p. for 24 h and challenged i.p. with LPS (20 mg/kg). Mice were monitored three times daily for 7 days.

### Statistical Analyses

All quantitative results are expressed as means ± SEM. One-way ANOVA was used to compare multiple groups followed by Tukey *post hoc* tests. Statistical analyses were conducted using GraphPad Prism Plus software, and a *p* < 0.05 was considered statistically significant.

## Results

### VitB6 Improves LPS-Induced Myocardial Damage

**FIGURE 1:**
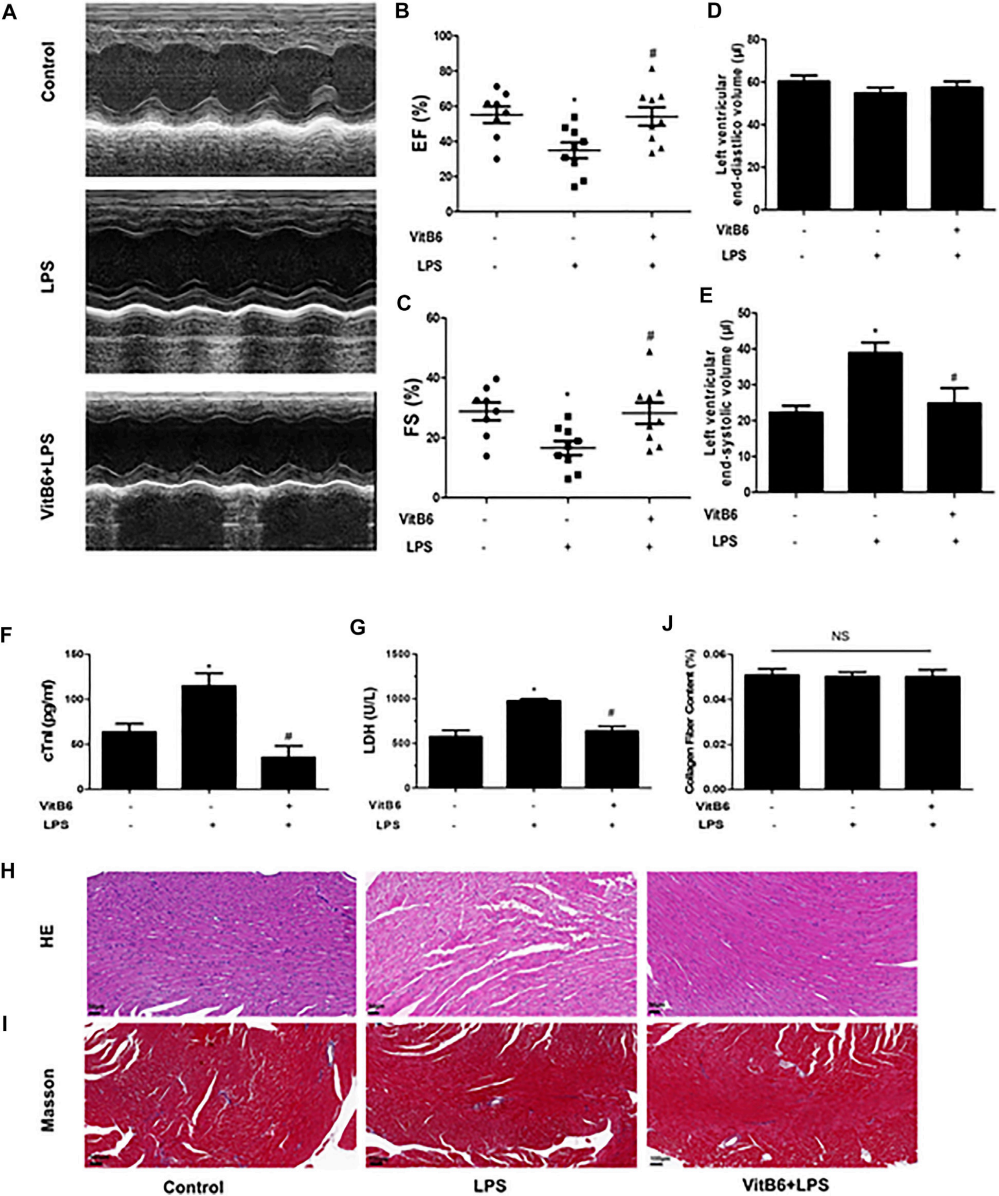
VitB6 improves LPS-induced myocardial damage. C57BL/6 mice were divided into control (*n* = 8), LPS (*n* = 9), and VitB6+LPS (*n* = 9) groups. Mice were pretreated with VitB6 (20 mg/kg) for 6 h and then treated with LPS (4 mg/kg) for 24 h. Before euthanasia, cardiac ultrasound was performed on mice (*n* = 8–9). Representative images of M-mode echogram of the heart **(A)**. The ejection fraction (EF) **(B)** and fractional shortening (FS) **(C)** were measured. The left ventricular end-diastolic volume **(D)** and the left end-systolic **(E)** were detected by ultrasound. Serum was isolated from blood samples and serum cTnI and LDH were tested using assay kits **(F** and **G)**. Myocardial tissue with H&E staining exhibits the morphology **(H)**. Masson stain was applied to explore the difference in collagen deposition **(I)**. Representative images are shown. *p < 0.05 vs. control group; ^*#*^ *p* < 0.05 vs. LPS group. Results are presented as means ± SEM.

Previous studies have demonstrated that VitB6 could improve myocardial ischemia (Pham et al., 2003; Kandzari et al., 2005). In the current study, to investigate VitB6 protective effects on LPS-induced myocardial injury, mice were pretreated with VitB6 for 6 h and then challenged with LPS (4 mg/kg). The EF and FS significantly decreased in LPS group mice compared with controls, but cardiac dysfunction improved in the VitB6+LPS group (Figures 1A–C). No differences were detected in the left ventricular end-diastolic volume between groups. However, the left ventricular end-systolic volume was reduced in the VitB6+LPS group (Figures 1D,E). Serum cTnI and LDH, which indicated myocardial injury appearance, increased in LPS-challenged mice compared with control and VitB6-pretreated groups (Figures 1F,G). Myocardial tissue morphology stained with H&E is shown in Figure 1H. The myofibrillar structure was normal in the control group, but LPS-challenged myocardial tissue appeared swollen and fractured. However, the VitB6+LPS group showed a mild swollen. LPS and VitB6 did not affect collagen deposition (Figures 1I,J).

### VitB6 Alleviates LPS-Induced Non-heme Iron Increase

**FIGURE 2:**
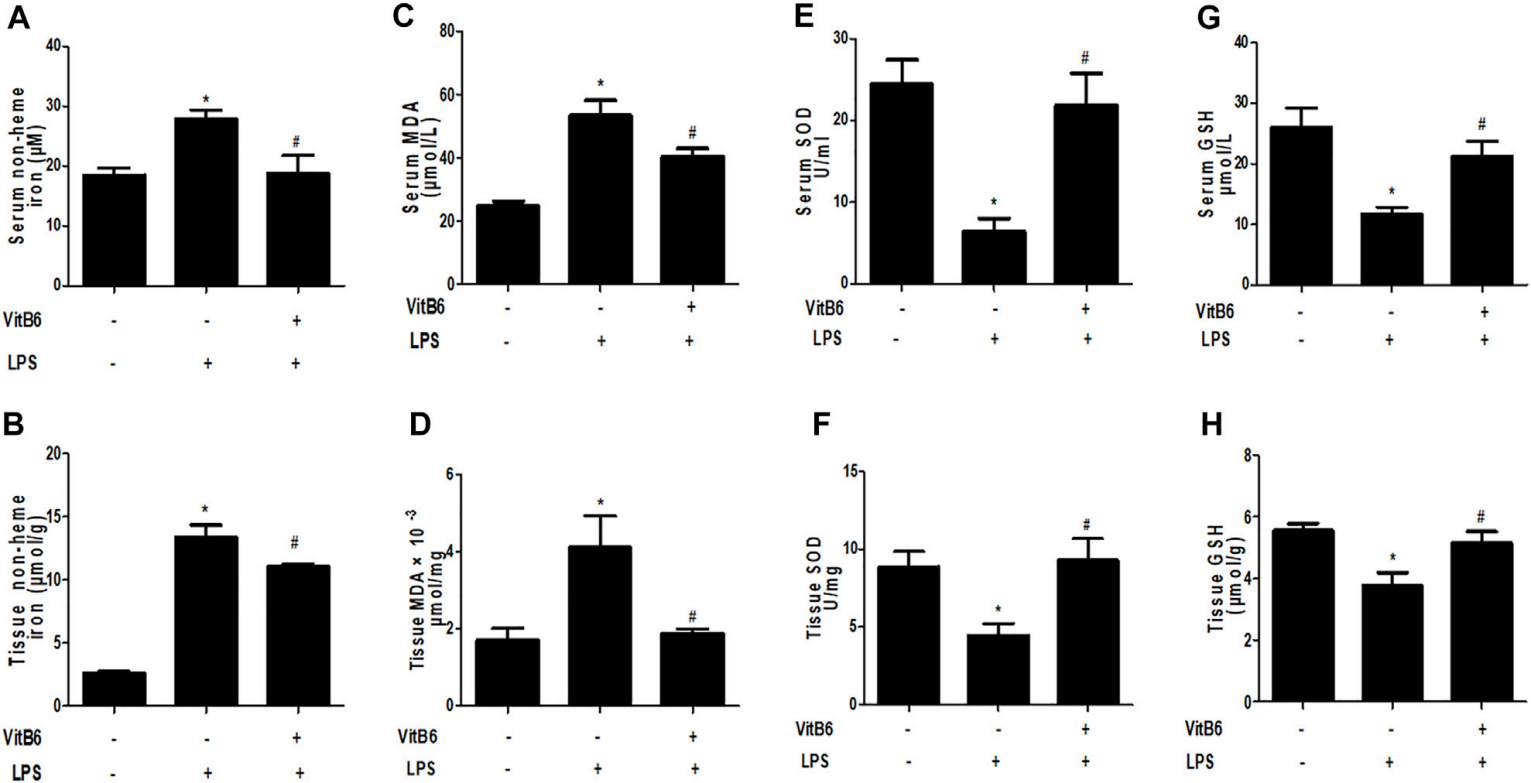
VitB6 eases LPS-induced increase in non-heme iron and suppresses LPS-induced oxidative stress and lipid peroxidation *in vivo*. C57BL/6 mice received PBS or ViitB6 (20 mg/kg) for 6 h and then LPS (4 mg/kg) for 24 h. Iron in serum **(A)** and myocardium **(B)** were assayed by a specific kit (*n* = 4–7, per group). Serum and myocardial tissue were also collected. MDA, SOD, and GSH levels *in vivo* of the three groups were assayed by specific kits **(C**–**H)**. *n* = 4–8, per group. *p < 0.05 vs. control group; ^*#*^ *p* < 0.05 vs. LPS group. Results are presented as means ± SEM.

Ferroptosis is iron-dependent and characterized by the accumulation of intracellular ROS and lipid peroxidation products (Dixon et al., 2012; Li et al., 2021). To explore the influences of LPS and VitB6 on non-heme iron, we measured iron levels in serum and myocardial tissue. We found that LPS-treated mice presented notably higher iron levels in serum and heart tissue compared with the control group. We also found that this trend was reversed by VitB6 in LPS-induced mice (Figures 2A,B). These results indicated that VitB6 might mediate iron metabolism.

### VitB6 Attenuates LPS-Induced Oxidative Stress and Lipid Peroxidation *in vivo*

MDA is one of the most important membrane lipid peroxidation products and impairs the activities of key mitochondrial enzymes, finally promoting aging (Yang et al., 2019). SOD is a crucial antioxidant enzyme that can scavenge free radicals (Yang et al., 2019). Glutathione (GSH)—a glycine, glutamic acid, and cysteine tripeptide—is a vital antioxidant that can protect hemoglobin from oxidation. In LPS-treated mice, MDA levels increased and were significantly attenuated by VitB6 (Figures 2C,D). In LPS-induced mice, SOD activity (Figures 2E,F) and GSH levels (Figures 2G,H) markedly decreased. However, this decrease could be reversed by VitB6. Nrf2, a crucial transcriptional activator for antioxidative responses (Ci et al., 2017), can be mediated by different stimuli, including LPS (Xu et al., 2018). To determine whether VitB6 affected Nrf2 expression in LPS-stimulated myocardial tissue, we pretreated mice with VitB6 followed by LPS. We found that VitB6 increased Nrf2 expression (Figure 6C). In addition, Nrf2 mRNA levels in the myocardium were consistent with its protein levels (Figure 6D). Altogether, these results demonstrated that VitB6 inhibit LPS-caused oxidative stress and peroxidation *via* Nrf2 activation.

### VitB6 ameliorates LPS-Induced Oxidative Stress and Lipid Peroxidation in H9C2 Cells

**FIGURE 3:**
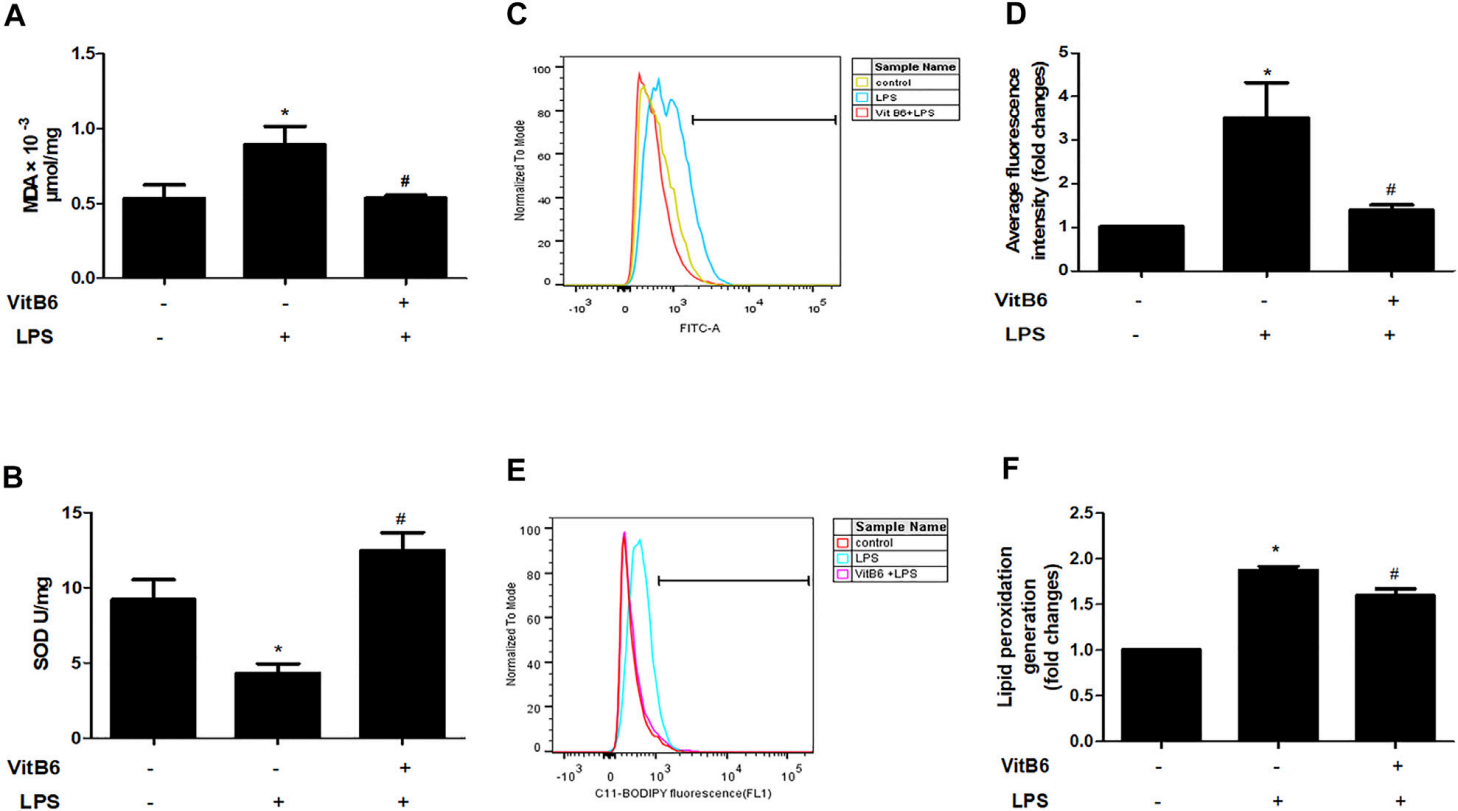
VitB6 alleviates LPS-induced oxidative stress and lipid peroxidation in H9C2 cells. The H9C2 cells were pretreated with VitB6 (500 μM) for 2 h and then treated with LPS (100 ng/m) for 24 h. MDA **(A)** and SOD **(B)** were measured by corresponding kits. ROS was assayed by flow cytometry and the representative flow curves are shown in **(C)** and the statistical result in **(D)**. Lipid peroxidation was detected by flow cytometry and the representative flow curves are shown in **(E)** and the statistical result in **(F)**. *n* = 4–5, per group. *p < 0.05 vs. control group; ^*#*^ *p* < 0.05 vs. LPS group. Results are presented as means ± SEM.

To further identify the alterations caused by LPS *in vitro*, we estimated SOD, ROS, and lipid peroxidation (lipid ROS) levels*.* Cells were pretreated with VitB6 for 2 h followed by LPS for 24 h. As predicted, in LPS-treated H9C2 cells, MDA, ROS, and lipid ROS levels increased, and SOD activity decreased. However, the above alterations were ameliorated when cells were exposed to VitB6 (Figures 3A–F). Therefore, these results are consistent with our *in vivo* results.

VitB6 inhibited erastin-induced ferroptosis *in vitro*.

Erastin is a small molecule that can trigger ferroptosis. To verify that VitB6 could suppress ferroptosis, H9C2 cells were pretreated with VitB6 for 2 h followed by erastin for 24 h. Erastin induced cells overt death and, in contrast, VitB6 suppressed erastin-induced cell death (Supplementary Figure S1). Thus, VitB6 inhibits ferroptosis induced by erastin as expected.

### VitB6 Regulates the Expression of Iron Regulatory Proteins in H9C2 Cells

**FIGURE 4:**
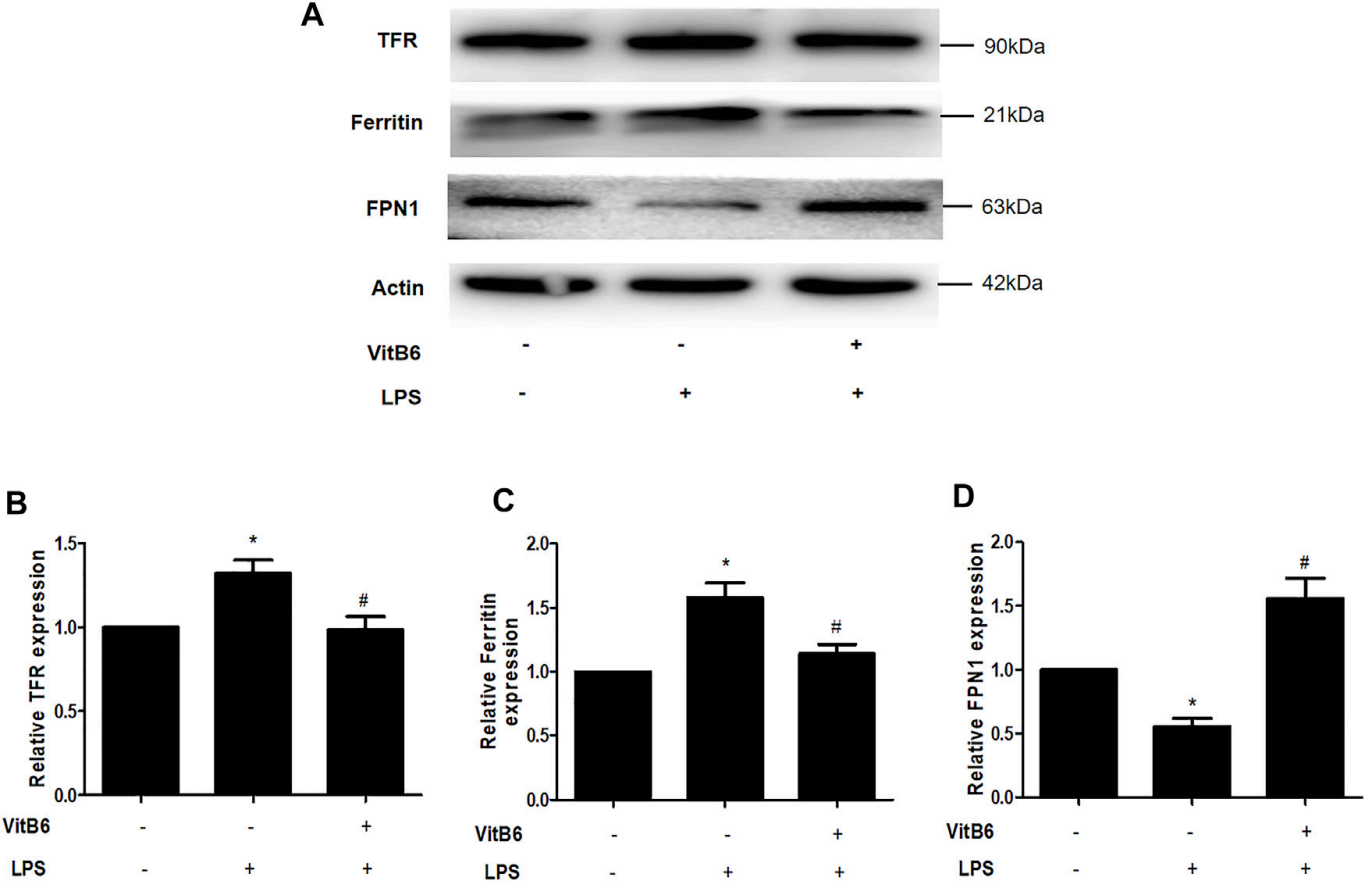
VitB6 regulates the expression of iron-regulated proteins *in vitro*. H9C2 cells were preconditioned with VitB6 (500 μM) for 2 h followed by LPS (100 ng/ml). The TFR, ferritin, and FPN1 levels in cell lysates were detected by Western blot and characteristic images are shown in **(A)**. The levels of these proteins are in **(B–D)**. *n* = 3–5, per group. *p < 0.05 vs. control group; ^*#*^ *p* < 0.05 vs. LPS group.

VitB6 decreased nonheme iron levels in serum and myocardial tissue LPS-induced *in vivo*. Iron homeostasis maintenance depends on the participation of different ferroregulatory proteins. For example, transferrin receptor (TFR) transports iron into cells and FPN1 exports iron out of cells (Gkouvatsos et al., 2012; Ward and Kaplan, 2012), thereby regulating cellular iron levels. Ferritin is an iron-binding protein and can be used to store iron (Huang et al., 2019). Thus, we explored the expression of iron regulatory proteins TFR, FPN1, and ferritin. Cells were pretreated with VitB6 for 2 h followed by LPS for 24 h. Then, lysates were subjected to Western blot. After LPS treatment, the levels of TFR and ferritin increased (Figures 4A–C), and the expression of FPN1 decreased (Figures 4A–D). In contrast, the synthesis of TFR, ferritin, and FPN1 was reversed after treatment with VitB6+LPS (Figures 4A–D). These results indicated that LPS may lead to an iron elevation in H9C2 cells, as necessary for ferroptosis.

### VitB6 Activates Nrf2 and Increases the Expression of Related Antioxidant Enzymes

**FIGURE 5:**
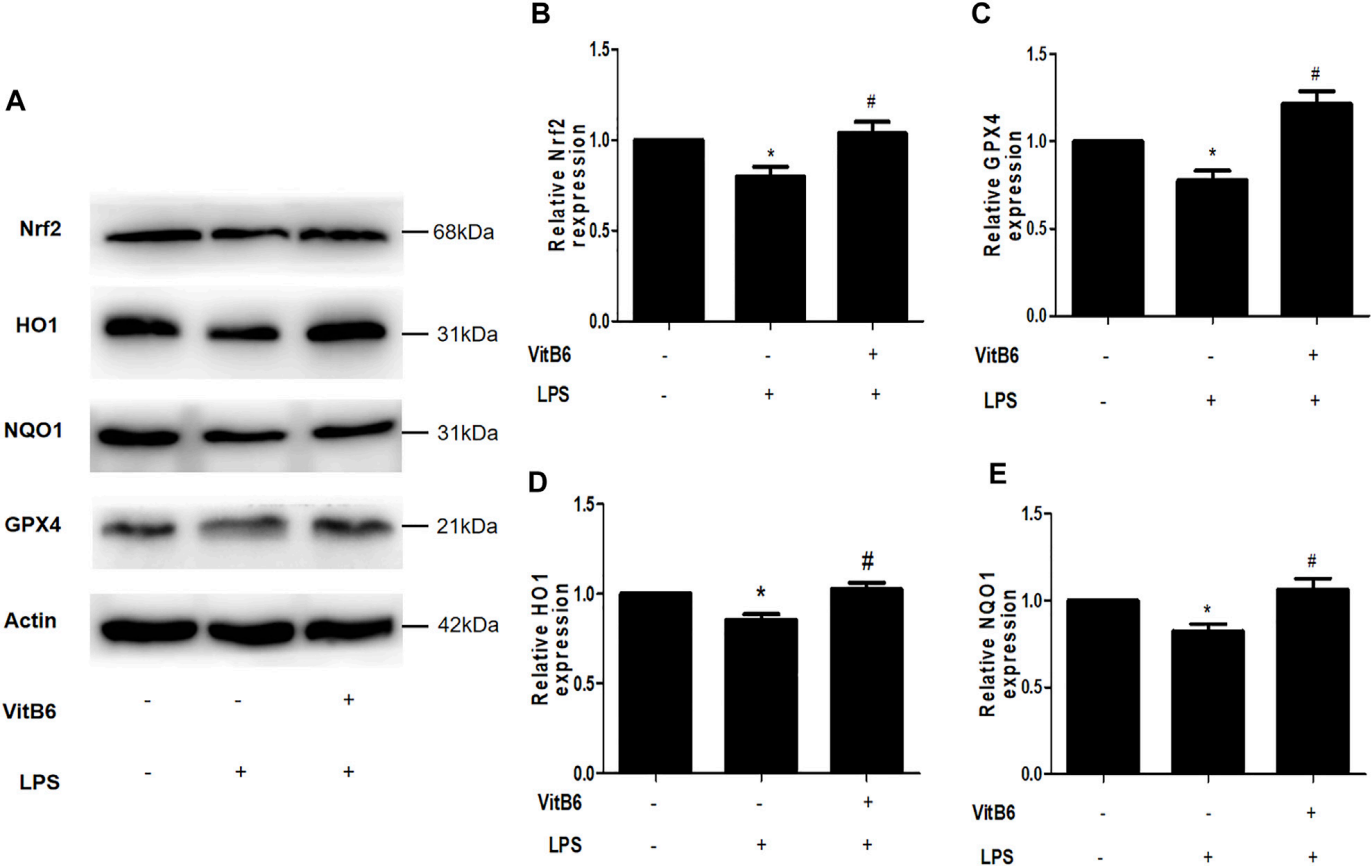
VitB6 upregulates the expression of Nrf2 and related antioxidant enzymes *in vitro*. H9C2 cells were pretreated with VitB6 (500 μM) for 2 h and then treated with LPS (100 ng/ml) for 24 h. Total cell lysates were subjected to Western blot to detect Nrf2, HO1, NQO1, and GPX4 levels. Representative images are shown in **(A)**. The levels of these proteins are in **(B–E)**. *n* = 3–5, per group. *p < 0.05 vs. control group; ^*#*^ *p* < 0.05 vs. LPS group.

Many studies have shown that Gpx4 inactivation can trigger ferroptosis (Ingold et al., 2018) and that upregulation of Nrf2 signaling can suppress ferroptosis and apoptosis in various diseases (Sun et al., 2016; Ishii et al., 2019). NQO1 and HO1 are crucial antioxidant-related enzymes and play a vital role in the Nrf2 signaling pathway (Ishii et al., 2000; Sun et al., 2016). To elucidate VitB6 effects on these proteins, we pretreated cells with VitB6 for 2 h followed by LPS for 24 h. Then, lysates were subjected to Western blot. The LPS treatment decreased Nrf2, Gpx4, NQO1, and HO1 expressions (Figures 5A–E). On the other hand, VitB6 significantly increased the expression of these proteins (Figure 5A). These results indicated that VitB6 altered the synthesis of proteins related to ferroptosis and apoptosis.

### VitB6 Alleviates Apoptosis in LPS-Induced Myocardial Tissue

**FIGURE 6:**
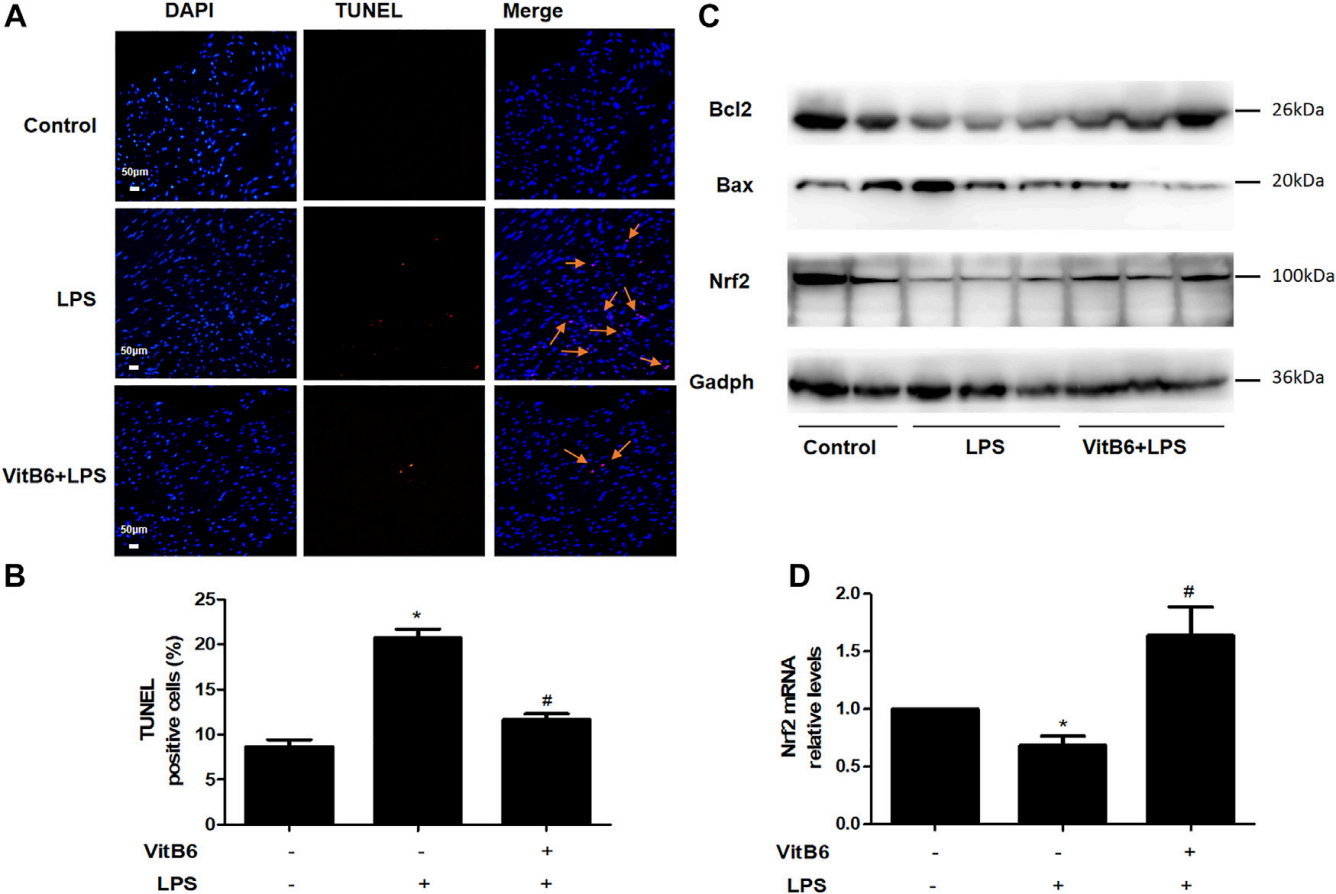
VitB6 suppresses LPS-induced apoptosis and increases Nrf2 expression *in vivo*. C57BL/6 mice were pretreated with PBS or VitB6 (20 mg/kg) for 6 h and then treated with LPS (4 mg/kg) for 24 h. The myocardium of mice was fixed in paraformaldehyde for almost 48 h and then subjected to TUNEL staining **(A**,**B)**. The Bcl2, Bax, and Nrf2 levels in myocardial tissue protein lysates were detected by Western blot **(C)**. Nrf2 mRNA levels **(D)** were assayed by real-time PCR. *n* = 3–6, per group. *p < 0.05 vs. control group; ^*#*^ *p* < 0.05 vs. LPS group. Results are presented as the means ± SEM.

Studies have reported that VitB6 can protect the intestinal epithelium from ionizing radiation-induced apoptosis (Thotala et al., 2009). Thus, we used the TUNEL assay to investigate whether VitB6 inhibited LPS-induced apoptosis. The number of TUNEL-positive cells increased followed by LPS stimulation. However, in VitB6 pretreated myocardium, the number of positive cells was reduced (Figures 6A,B). In addition, LPS increased Bax protein levels and decreased Bcl2 expression of myocardial tissue, but VitB6 reversed this Bax/Bcl2 trend (Figure 6C). These data revealed that VitB6 can regulate LPS-activated intrinsic apoptotic pathway.

### VitB6 Suppresses LPS-Induced Apoptosis in H9C2 Cells

**FIGURE 7:**
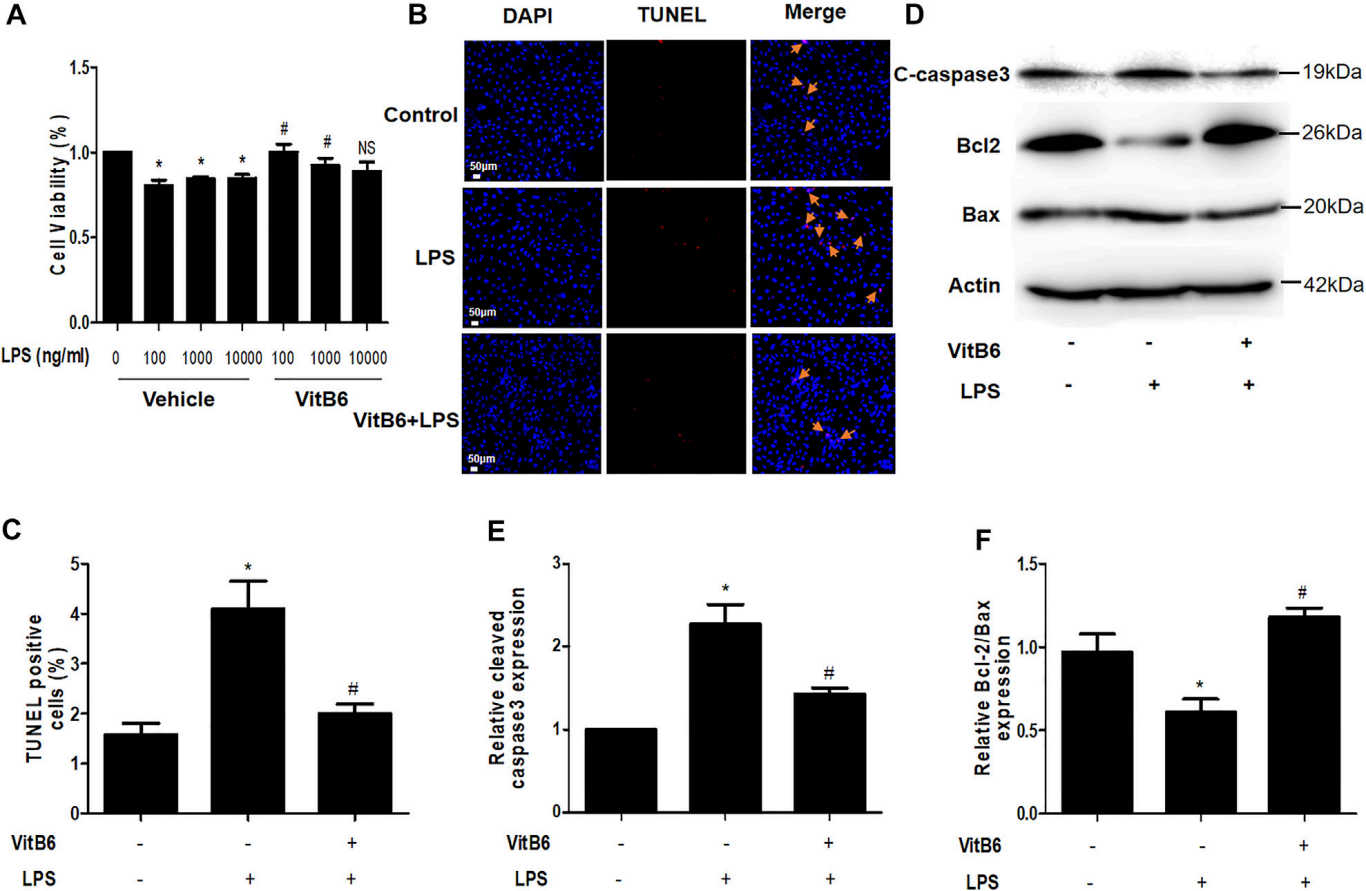
VitB6 alleviates LPS-induced apoptosis *in vitro*. The LPS-induced cytotoxicity and VitB6 effect on LPS-induced cytotoxicity were measured in H9C2 cells after 24 h incubation. Cells seeded in a 96-well plate were pretreated with VitB6 (500 μM) and then treated with LPS (0, 100, 1,000, and 10,000 ng/ml) for 24 h. Cell viability was assayed by Cell Counting Kits, and the cell viability percentage is shown in **(A)**. H9C2 cells were preconditioned with VitB6 (500 μM) for 2 h followed by LPS (100 ng/ml) for 24 h. **(B**, **C)** Cells fixed in paraformaldehyde TUNEL stained in **(B)** and quantified in **(C)**. *n* = 7, per group. *p < 0.05 vs. control group; ^*#*^ *p* < 0.05 vs. LPS group. **(D–F)** C-caspase3, Bcl2, and Bax expression levels in treated cell lysates assayed by Western blot. The blot is representative of at least three independent experiments. *n* = 3–4 per group. *p < 0.05 vs. control group; ^*#*^ *p* < 0.05 vs. LPS group. NS, no significance.

Cells seeded in 96-well plates were preprocessed with VitB6 (500 μM) or PBS for 2 h and then treated with different LPS concentrations for 24 h. Cell viability was assayed by Cell Counting Kit-8, and the cell viability percentage is depicted in Figure 7A. We observed that VitB6 reduced LPS-induced cell death at 100 and 1,000 ng/ml. To further investigate the VitB6 *in vitro* effects on apoptosis, cells were pretreated with VitB6 (500 μM) for 2 h and then stimulated with LPS (100 ng/ml) for 24 h. The TUNEL kit was used to elucidate the effect of VitB6 on LPS-induced cells. TUNEL-positive cells were significantly increased followed by LPS, and the numbers of TUNEL-positive cells of VitB6 pretreated lessened visibly. In response to LPS stimuli, the expression levels of apoptosis-related proteins, cleaved caspase3, and Bax increased but Bcl2 reduced. Pretreatment with VitB6 effectually repaired these alterations (Figures 7D–F).

### VitB6 Improves Mitochondrial Injury in LPS-Treated H9C2 Cells

Apoptosis is closely related to mitochondrial injury. The mitochondrial membrane of H9C2 cells in the control group was intact, including clear mitochondrial cristae (Supplementary Figure S2A). However, mitochondria swelled and its inner crest ruptured, and even disappeared, after LPS exposure (Supplementary Figure S2B). VitB6 improved some LPS-induced morphological alterations of mitochondria structure, but they remained a bit swollen (Supplementary Figure S2C). These outcomes showed that VitB6 can protect mitochondria from LPS-induced injury.

*In vivo*, Fer-1 and Emricasan mimicked pharmacological effects of VitB6 decreasing the mortality of LPS-induced mice.

We hypothesized that LPS may induce ferroptosis and apoptosis. Thus, we preconditioned mice with Fer-1 (2 mg/kg) (*n* = 14) and emricasan (2.5 mg/kg) (n = 5) for 24 h followed by LPS (20 mg/kg) and monitored them three times daily for over 7 days to further confirm this hypothesis. The survival percentages of mice preconditioned with Fer-1 and emricasan were higher than the LPS group (*n* = 15) (Supplementary Figure S3C). The protocol is depicted in Supplementary Figure S3A, B. VitB6 improve the survival percentages of mice stimulated with LPS (*n* = 15) (Supplementary Figure S3C).

## Discussion

In this study, we demonstrated that VitB6 improved LPS-induced myocardial dysfunction. LPS-induced systemic inflammation seriously damaged myocardial structure and function (Tzanavari et al., 2016; Luo et al., 2020). We found that LPS compromised the related function indexes in echocardiography and serum (Figures 1A–C,E–G). We showed that VitB6 pretreatment for 6 h followed by LPS administration normalized the evaluated parameters. Moreover, H&E stainings showed that LPS administration led to cardiac structure disorder (Figure 1H).

Our main found is that VitB6 plays a crucial role in defending the myocardium against LPS-induced damage. It is well known that VitB6 supplementation can be used in the treatment of, for example, rheumatoid arthritis, Alzheimer’s disease, and stroke (Marchant et al., 2012; Gaieski et al., 2013). Recently, several studies have demonstrated that VitB6 improved the I/R-induced myocardial injury (Dhalla et al., 2013; Surendran et al., 2019). Here, we indicated that VitB6 lessens LPS-induced myocardial injury ferroptosis and apoptosis modulation. In addition, VitB6 alleviated lipid peroxidation and reduced LPS-induced oxidative stress consistent with previous studies regarding H_2_O_2_-induced monocytes and hyperhomocysteinemia (Kannan and Jain, 2004; Hsu et al., 2015; Molina-López et al., 2016).

Mechanistically, we showed that VitB6 contributed to LPS-caused ferroptosis and apoptosis suppression. Yogendra Singh et al. considered that the ferroptosis stress triggered cell death, and the transsulfuration pathway was regulated by homocysteine during COVID-19 infections (Singh et al., 2020). PLP, the active form of VitB6, regulates the transsulfuration pathway (Shan et al., 2020). However, there was no direct proof demonstrating that VitB6 can suppress ferroptosis. First, we demonstrated that VitB6 can alleviate LPS-induced oxidative stress and lipid peroxidation that can trigger ferroptosis *in vivo* and *in vitro*. Second, VitB6 suppressed the LPS-induced apoptosis in cells and mice. Third, VitB6 restrained the release of non-heme iron *in vivo* and improved mitochondrial damage. Importantly, LPS inhibits the expression of Nrf2, NQO1, HO1, and Gpx4 but VitB6 reversed this effect. Moreover, LPS upregulates TFR and ferritin levels and downregulates FPN1 expression, reversed by VitB6.

In this study, VitB6 supplementation could inhibit the generation of lipid peroxidation and ROS, consistent with previous studies (Kannan and Jain, 2004; Ardestani et al., 2008; Molina-López et al., 2016). The generation of ROS oxidative stress and GSH activation suppression is closely related to apoptosis and ferroptosis (Kerr et al., 1972; Yang et al., 2014). GSH depletion results in ROS accumulation (Chisté et al., 2014). In addition, the homocysteine conversion to cysteine by cystathionine-*β*-synthase is VitB6-dependent (Nijhout et al., 2009). Cysteine increased GSH generation and further lowered LPS-induced ROS accumulation, catalyzed by Gpx4 (Lewerenz et al., 2013). Erastin, a cystine/glutamate antiporter (system x_c_^−^) inhibitor, could block GSH activation, resulting in ROS accumulation and ferroptosis. In our study, LPS-treated cells and mice showed a similar effect with erastin, but the effect was not as strong. In addition, VitB6 reversed the effect of LPS as far as possible.

Our results are in agreement with previous studies, in which LPS downregulated antioxidant regulatory proteins Nrf2, NQO1, HO1, and Gpx4 (Hyung et al., 2016). In contrast, VitB6 distinctly upregulated the levels of these proteins. Nrf2 was reported to be the center of the upregulation of antioxidant enzymes expression, which could activate HO1, NQO1, and Gpx4 (Tang et al., 2019). The activation of Nrf2/HO1 is associated with ferroptosis and apoptosis (Kannan and Jain, 2004; Tan et al., 2021). Transferrin, an iron-transport protein, transports iron to the TFR 1 at the surface of the cells (Ward and Kaplan, 2012). In addition, ferroportin, an iron efflux transporter, releases iron outside cells (Dhalla et al., 2013). In this study, LPS increased the TFR and ferritin levels and decreased FPN1 generation, thereby increasing intracellular iron levels. Nevertheless, VitB6 rescues this alteration.

Overall, in the present study, we demonstrated a novel function of VitB6 in combat of LPS-induced myocardial injury. Moderate amount of VitB6 presented therapeutic properties against myocardial damage caused by sepsis. The function of VitB6 in LPS-induced ferroptosis and apoptosis showed the potential of ferroptosis or apoptosis inhibitors as new drugs for sepsis-caused myocardial damage treatment.

## Data Availability

The original contributions presented in the study are included in the article/Supplementary Material, further inquiries can be directed to the corresponding author.
