# Supplementary figures and images for: Vitamin B6 Alleviates Lipopolysaccharide-induced Myocardial Injury by Ferroptosis and Apoptosis Regulation
Source: Front Pharmacol. 2021 Dec 24;12:766820. doi: 10.3389/fphar.2021.766820 (PMC8740299; doi:10.3389/fphar.2021.766820)

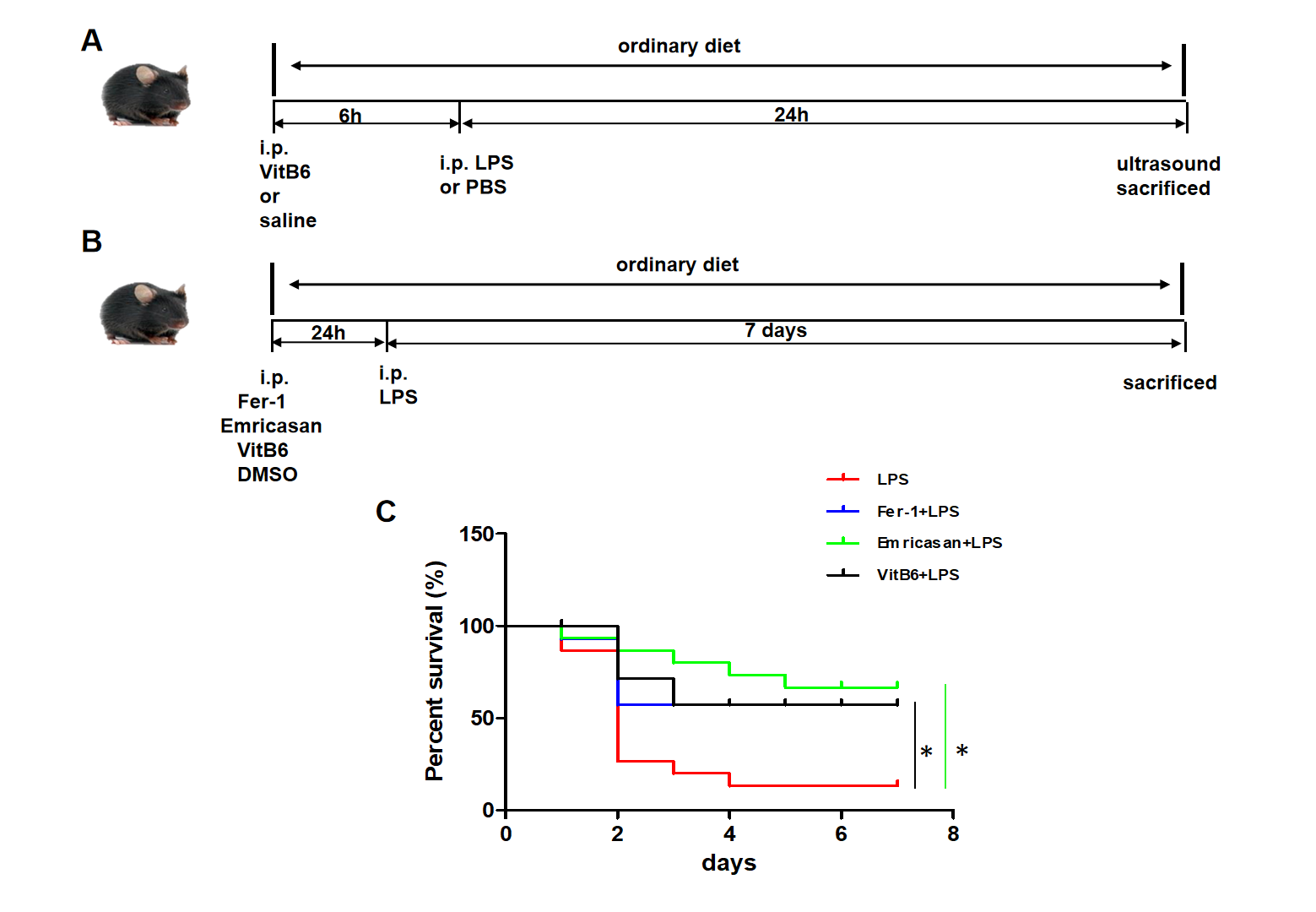

Supplement: Supplementary file 1 [file Image3.TIF]

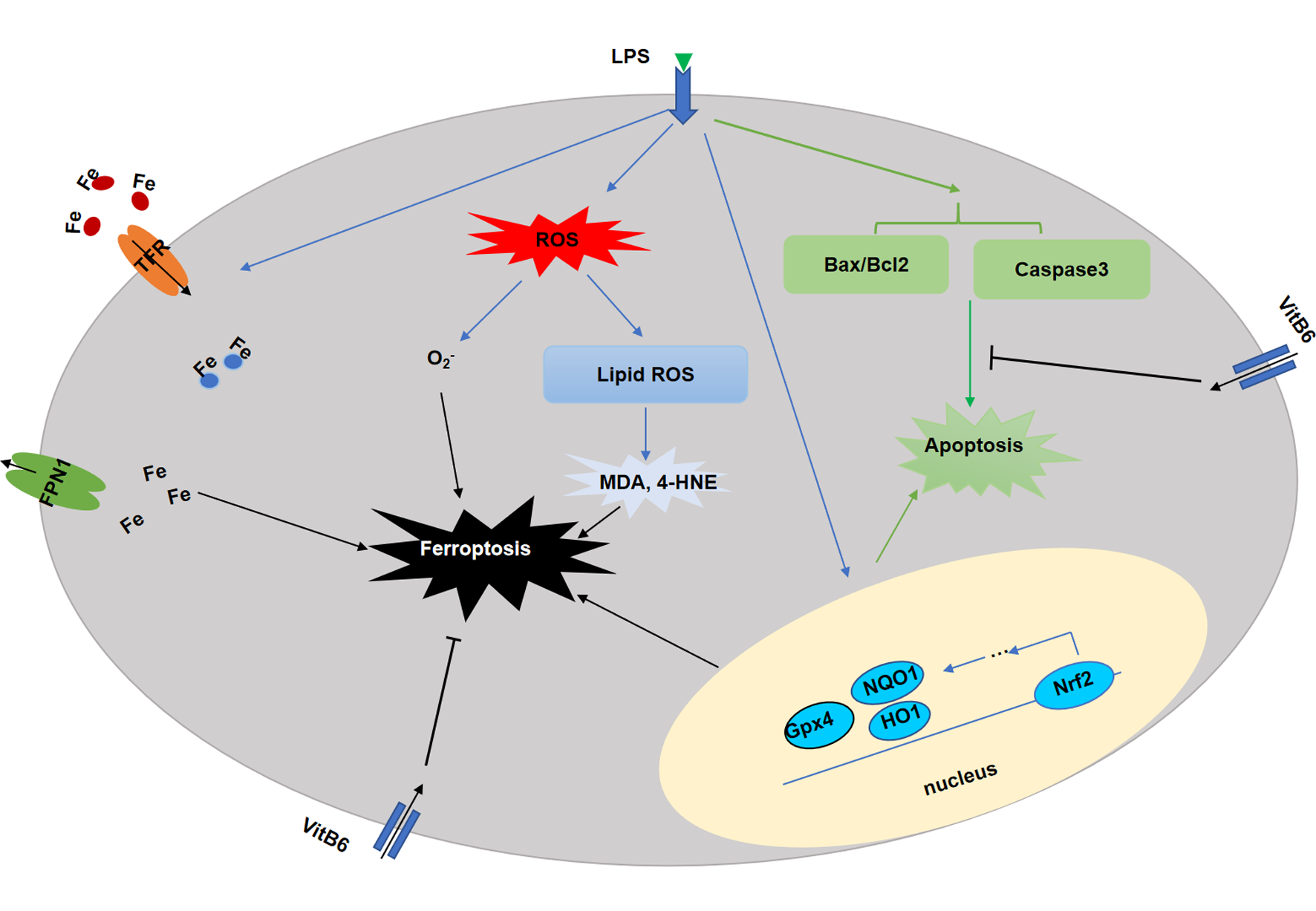

Supplement: Supplementary file 2 [file Image4.TIF]

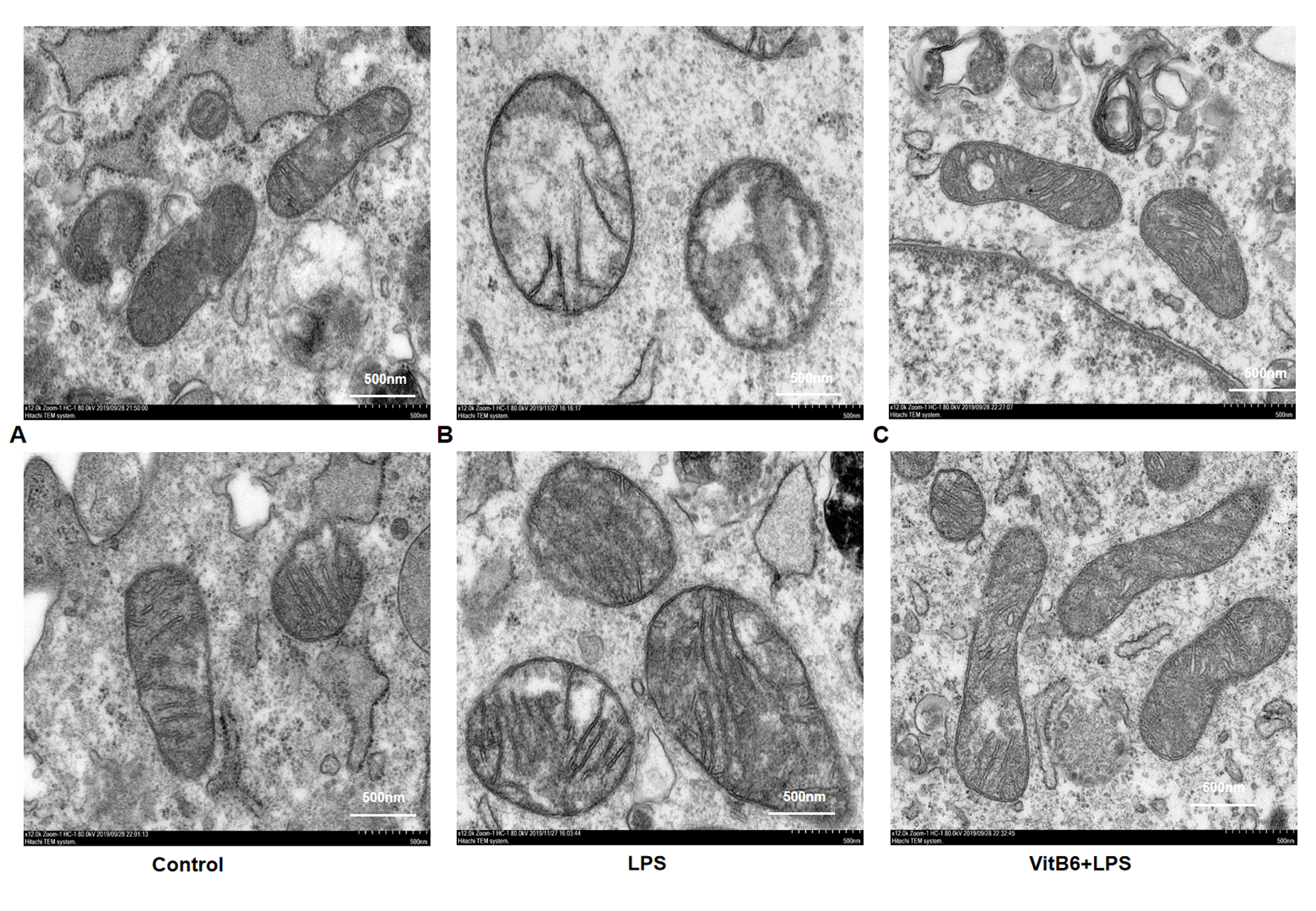

Supplement: Supplementary file 3 [file Image2.TIF]

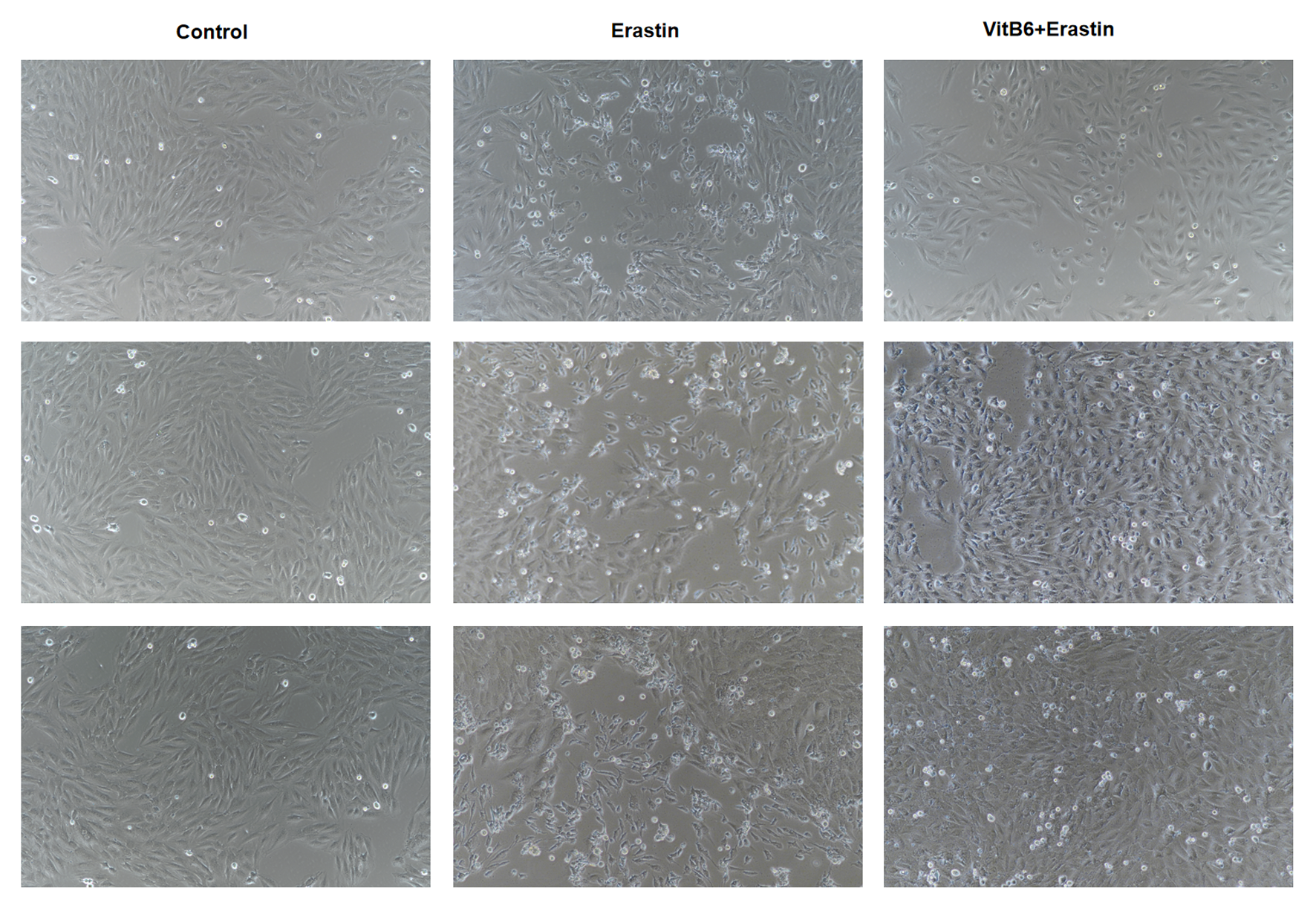

Supplement: Supplementary file 4 [file Image1.TIF]
